# Comparison by Age of the Local Interferon Response to SARS-CoV-2 Suggests a Role for IFN-ε and -ω

**DOI:** 10.3389/fimmu.2022.873232

**Published:** 2022-07-12

**Authors:** Alessandra Pierangeli, Massimo Gentile, Giuseppe Oliveto, Federica Frasca, Leonardo Sorrentino, Luigi Matera, Raffaella Nenna, Agnese Viscido, Matteo Fracella, Laura Petrarca, Gabriella D’Ettorre, Giancarlo Ceccarelli, Fabio Midulla, Guido Antonelli, Carolina Scagnolari

**Affiliations:** ^1^ Virology Laboratory, Department of Molecular Medicine, “Sapienza” University, Rome, Italy; ^2^ Department of Maternal, Infantile and Urological Sciences, “Sapienza” University, Rome, Italy; ^3^ Department of Public Health and Infectious Diseases, “Sapienza” University, Rome, Italy

**Keywords:** SARS-CoV-2, children, innate immunity, type I interferon, IFN-ε, IFN-ω

## Abstract

Children generally develop a mild disease after SARS-CoV-2 infection whereas older adults are at risk of developing severe COVID-19. Recent transcriptomic analysis showed pre-activated innate immunity in children, resulting in a more effective anti-SARS-CoV-2 response upon infection. To further characterize age-related differences, we studied type I and III interferon (IFN) response in SARS-CoV-2 infected and non-infected individuals of different ages. Specifically, levels of expression of type I (IFN-α, -β, -ε and -ω), type III (IFN-λ1, -λ2 and -λ3) IFNs and of the IFN-stimulated genes, ISG15 and ISG56 were quantified in nasopharyngeal cells from diagnostic swabs. Basal transcription of type I/III IFN genes was highest among children and decreased with age. Among SARS-CoV-2-infected individuals, only IFN-ε and -ω levels were significantly higher in children and young adults whereas ISGs were overexpressed in infected adults. The occurrence of symptoms in children and the need for hospitalization in adults were associated to higher transcription of several IFN genes. Starting from a pre-activated transcription level, the expression of type I and III IFNs was not highly up-regulated in children upon SARS-CoV-2 infection; young adults activated IFNs’ transcription at intermediate levels whereas older adults were characterized by higher ISGs and lower IFN-ε and -ω relative expression levels. Overall, our findings contribute to recognize components of a protective IFN response as a function of age, in the context of SARS-CoV-2 infection.

## Introduction

From December 2019 up to now, the novel coronavirus named SARS-CoV-2 has caused a huge impact on health worldwide due to life-threatening COVID-19 disease. Risk factors predisposing to severe COVID-19 are male sex, the presence of comorbidities and an older age, whereas children commonly develop a mild disease after SARS-CoV-2 infection (1). The mechanisms that protect the younger from severe COVID-19 disease are yet to be defined; reasons may be a low expression of the ACE2 receptor, trained immunity, a higher capacity to regenerate cells after viral damage but also a strong and effective innate and/or adaptive immune response to SARS-CoV-2 (1–3). SARS-CoV-2 replication starts in nasopharyngeal mucosal cells and recognition of viral molecular patterns leads to a rapid activation of antiviral and inflammatory responses (4, 5). The antiviral response is largely mediated by the transcription of Interferon (IFN) genes, in particular the type I (IFN-α, -β) and type III (IFN-λ1-3) that are the most important IFNs produced by many cell types. Once the IFNs are secreted from infected cells and bind to specific receptors, their signaling upregulates the expression of numerous IFN-stimulated genes (ISGs). In infected cells, SARS-CoV-2 non-structural proteins are able to inhibit the innate immune receptor signaling and IFN production (6); on the other hand, SARS-CoV-2 is highly sensitive to type I/III IFNs when exogenously administered (7, 8). In patients, the key protective role of the IFN-response was confirmed by genetic studies showing that mutations in genes of the type I IFN pathway are far more frequent in patients with severe COVID-19 than in those with a mild disease (9). Moreover, circulating auto-antibodies (Abs) against type 1 IFNs were detected in around 10% of critically ill COVID-19 patients (10). While a fraction of severely COVID-19 patients is highly suppressed in their IFN production and activity due to genetic defects, the presence of anti-IFN auto-Abs or other causes (9–12), on the other hand, delayed and disproportionate IFN responses are involved in the progression to severe disease (4, 13, 14). Thus, to achieve control of SARS-CoV-2 infection, a fast and effective mucosal innate response appears crucial (15), as seen in adults that developed a mild COVID-19 (14–17). Consistent with this notion, children, which usually develop asymptomatic or very mild COVID-19, present a more robust early innate immune response in comparison with adults (18, 19). In particular, a higher expression of genes associated with IFN signaling was found in SARS-CoV-2 infected children with respect to adults, not stratified by age (18). Loske et al. found that basal levels of several Pattern Recognition Receptors and Interferon-stimulated genes (ISG) were pre-activated in children and that, upon SARS-CoV-2 infection, their expression reached higher levels in children compared to adults (19); in this study, the expression of the IFN coding genes could not be detected. Contrastingly, Gilbert et al. measured the mRNA levels of type I (IFN-α and -β) and type III (IFN-λ1-3) IFNs, reporting that IFN α and -β transcript levels were induced by SARS-CoV-2 infection, reaching more elevated levels in the adult/elderly with respect to the pediatric age in which only IFN- λ1 was comparatively higher (20).

In order to determine which IFNs are activated during an effective anti-SARS-CoV-2 immune response in the upper respiratory tract, we sought to compare this response among different age groups, with respect to negative controls of the same age. Specifically, we measured mRNA expression levels of genes encoding type I (including the less studied IFN-ε and -ω) and type III IFNs, and IFN-stimulated genes in nasopharyngeal cells from subjects of ages ranging from 3 to 90 years, that were evaluated also for symptoms and the need for hospitalization.

## Materials and Methods

### Study Subjects

Children/adolescents attending a pediatric ambulatory clinic of the “Umberto I”, Sapienza University Hospital in Rome, to performSARS-CoV-2 molecular tests, were enrolled (September-December 2020) after parent’s written consent (N=47). Children/adolescents were tested because of a previous contact with a SARS-CoV-2 positive case within their family or at school, or for the occurrence of respiratory symptoms. Moreover, medicine students and post-graduates practicing in the pediatric clinic were tested in the same period. Adults attending the “Umberto I” Emergency Departments (ED) were recruited during two periods (March-May 2020 and September-December 2020); they were health workers attending routinely testing for SARS-CoV-2 infection or subjects presenting to the ED with fever and/or respiratory symptoms. A total of 145 adults (>16 years of age) were enrolled following informed consent. SARS-CoV-2 detection was performed in the same virology diagnostic laboratory, from nasopharyngeal (NP) swabs, using RealStar^®^ SARS-CoV-2 RT-PCR Kit 1.0 (Altona Diagnostics, Germany), targeting E and S viral genes. All SARS-CoV-2 positive patients were infected with the original D614G SARS-CoV-2 strain that was the only one circulating until late December 2020 when the Alpha variant was firstly detected in Italy. For subject tested at the ED, the threshold cycle (Ct) value from the diagnostic RT-PCR was obtained. Residual NP samples were stored for up to 3 hours at 4°C, then centrifuged, pellets added with guanidine isothiocyanate reagents and stored at -80 C; they were included in the study as cases if tested positive for SARS-CoV-2 or controls if negative. The occurrence of symptoms was reported by children’s parents and self-reported by adolescents and adults. Children/adolescents were categorized in symptomatic (those presenting one or more of the following symptoms: fever, cough, sore throat, dyspnea, ageusia, anosmia, vomiting, diarrhea) or asymptomatic. Contrastingly, nearly all SARS-CoV-2 positive adults presented with COVID-19 related symptoms and were retrospectively categorized on the base of the need for hospitalization. Demographic data, SARS-CoV-2 Ct values, the occurrence of symptoms, and the need for hospitalization were recorded de-identified. The study was approved by the Institutional Review board and the Ethics Committee (Policlinico Umberto I Hospital, Sapienza, University of Rome, Rif. 5836, Prot. 0690/2021).

### Gene Expression Measurement

De-frozen residual NP cells were centrifuged and pellets used for total RNA extraction using RNA-extraction kits (Norgen Biotek Corporation, Canada), 2 µl of purified RNA was quantified on a NanoDrop Spectrophotometer (Thermo Fisher Scientific, Rome, Italy) to determine concentration and purity. Reverse transcription was performed on 300 ng of purified RNA using the High Capacity cDNA Archive Kit (Applied Biosystems, Monza, Italy). From cDNA, the expression levels of genes coding for IFNs type I (IFN-α, -β, -ε and -ω) and type III (IFN-λ1, -λ2 and -λ3) and for ISG15 and ISG56, well-known markers of type I/III IFNs’ activation, previously analysed in SARS-CoV-2 infections (11, 17, 21), were measured by quantitative Reverse Transcription-Real time PCR assays. Briefly, IFN genes transcripts were detected in co-amplification with the beta-glucuronidase (*GUS*) invariant gene, using the target specific primers and hydrolysis probes listed in our previous study (21), with the addition of primers and probes targeting IFN-ε (Hs.PT.58.4812867.g) and IFN-ω (Hs.PT.20160308.g) purchased from IDT (Integrated DNA Technologies, Coralville, Iowa, US). Transcript level of each gene was calculated using the threshold cycle (Ct) relative quantification to the GUS Ct of the same sample (the 2^−ΔCt^ method). A gene transcript was considered not detectable when the Ct value was > 40 and was assigned a value of one tenth of the limit of detection. Residual cDNAs from SARS-CoV-2 negative samples were tested for the presence of 13 other respiratory viruses (influenza viruses A and B, respiratory syncytial virus, the human CoVs: OC43, 229E, NL63 and HKU1, adenovirus, rhinovirus, parainfluenza viruses 1-3, metapneumovirus), using a panel of home-made PCRs (22).

### Statistical Analysis

For categorical variables (sex, age group, occurrence of symptoms, need for hospitalization), Pearson’s Chi square was used to test the statistical difference in proportion among independent groups. Age in years, mRNA expression values (non-normally distributed data) were compared using the non-parametric Kruskal–Wallis (KW) test among three or more independent groups or using the non-parametric Mann–Whitney test between two groups with Bonferroni correction for multiple comparisons. The Jonckheere-Terpstra (JT) test, a rank-based nonparametric test, was also used for determine significant trends in gene expression levels among the ordered age groups. Spearman’s rho coefficient was calculated to assess the correlation between age and expression level of the studied genes. The significance was fixed at the 5% level; the analysis was performed with SPSS v.27.0 for Windows.

## Results

**Figure 1:**
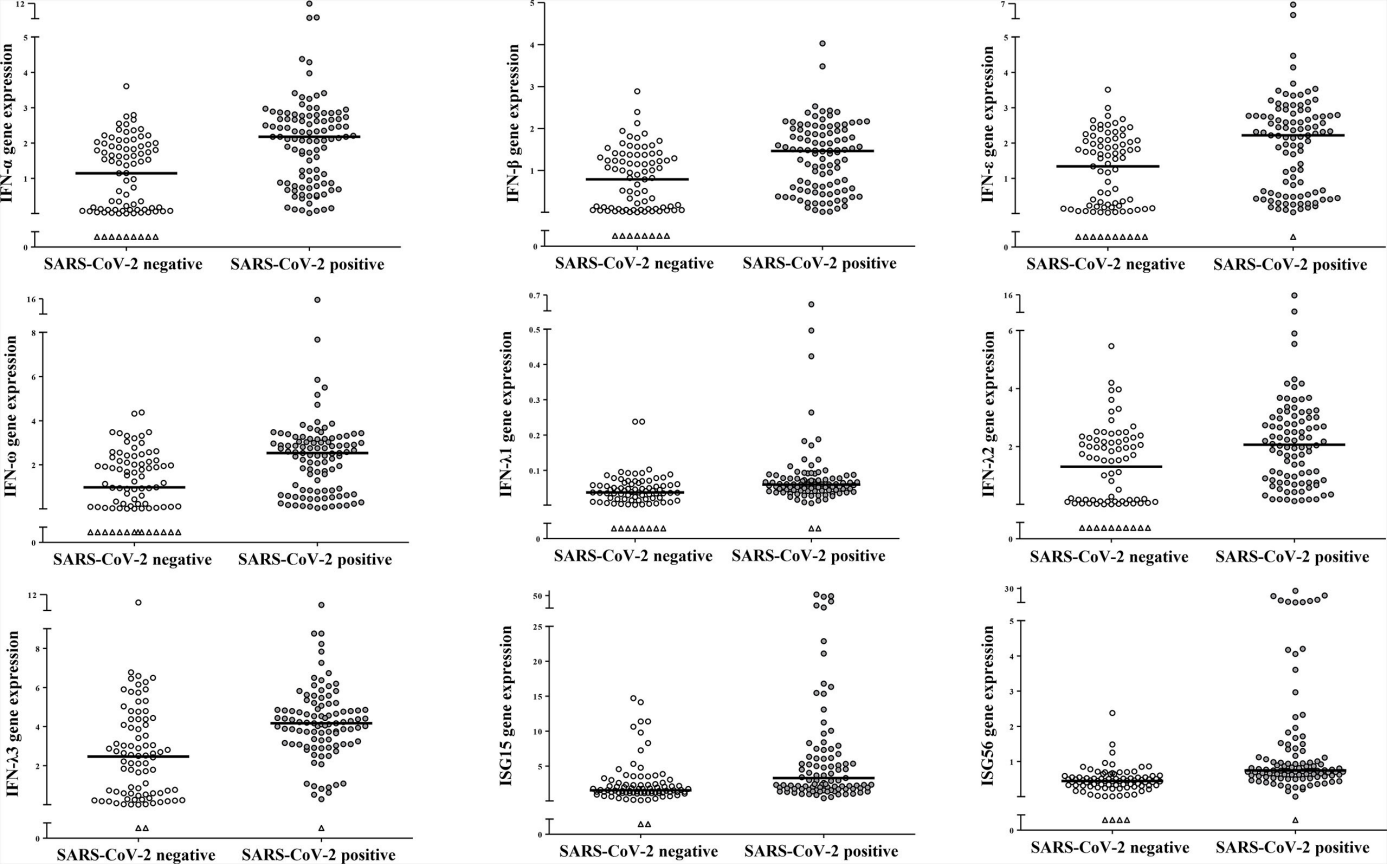
SARS-CoV-2 infection induced activation of IFN genes in nasopharyngeal cells. Relative mRNA expression values (y-axis), calculated using the threshold cycle relative quantification (2^-ΔCt^), are reported for each gene; values are depicted as open circles for SARS-CoV-2 negative- and as filled circles for SARS-CoV-2 positive samples, triangles indicate samples with Ct values >40. Horizontal lines indicate the median value of the group indicated below the X-axis. All p-values, calculated using Mann-Whitney tests for comparison of each gene between SARS-CoV-2 negative and positive samples, are <0.001.

Out of 192 individuals (114 males and 78 females) enrolled in this study, 106 tested positive for SARS-CoV-2 RNA and 86 resulted negative to SARS-CoV-2 and to the other respiratory viruses. Levels of expression of genes coding for type I (IFN-α, -β, -ε and -ω), type III (IFNs λ1-3) and for ISG15 and ISG56 were measured in 192 NP samples. Transcript levels of all study genes were significantly higher levels in the SARS-CoV-2 positive- than in the negative- samples (p<0.001 for comparisons of all genes between the two groups; **Figure 1**). Males and females had comparable gene expression levels when considering all samples together or when comparing by sex SARS-CoV-2 negative and positive-samples separately (data not shown). To understand the impact of age on IFN genes expression during SARS-CoV-2 infections, samples were stratified in age groups: children up to 16 years of age (y), young adults (17-40 y), middle-aged adults (41-60 y), older adults (>60 y) and on the base of SARS-CoV-2 negative- or -positive test. Demographic and other data of enrolled subjects stratified by age groups are reported in **Table 1**. Median and interquartile range values for each study gene in the 8 groups are shown in **Table 2**.

**Table 1:** Demographic and virologic data of study subjects (N=192) stratified by age groups.

	Female/Male(Female %)	Age ^a^	SARS-CoV-2Ct value^b^	Symptoms	Hospitalized
**SARS-CoV-2 Neg <16y (n=21)**	8/13 (38.1%)	8.9 ± 3.4	NA^c^	NA	NA
**SARS-CoV-2 Pos <16y (n=26)**	9/17 (34.6%)	10.5 ± 3.4	NR^d^	15/26 (57.7%)**	0/26 (0%)**
**SARS-CoV-2 Neg 16-40y (n=21)**	13/8 (61.9%)	28.9 ± 6.9	NA	NA	NA
**SARS-CoV-2 Pos 16-40y (n=24)**	12/12 (50.0%)	27.1 ± 7.4	27.6 ± 6.2	21/24 (87.5%)**	7/24 (29.2%)**
**SARS-CoV-2 Neg 41-60y (n=25)**	11/14 (44.0%)	50.5 ± 5.2	NA	NA	NA
**SARS-CoV-2 Pos 41-60y (n=28)**	8/20 (28.6%)	53.1 ± 4.4	27.9 ± 6.6	28/28 (100%)**	24/28 (85.7%)**
**SARS-CoV-2 Neg >60y (n=19)**	8/11 (42.1%)	64.5 ± 2.5*	NA	NA	NA
**SARS-CoV-2 Pos >60y (n=28)**	9/19 (32.1%)	73.3 ± 8.5*	26.7 ± 5.3	28/28 (100%)**	22/28 (78.6%)**

a Age in years (y) is expressed as mean ± standard deviation of each group.

b The diagnostic test Ct value is expressed as mean ± standard deviation in adults’ groups.

c NA, not applicable.

d NR, not reported. *p < 0.01, for age comparison between SARS-CoV-2 negative vs positive older adults; **p < 0.001, for comparisons among the SARS-CoV-2 positive age groups.

**Table 2:** Gene expression results of study samples (N=192) stratified by SARS-CoV-2 infection and age group.

	SARS-CoV-2 negative samples by age group				p value^b^	SARS-CoV-2 positive samples by age group				p value^b^
Gene ^a^	<16y	16-40y	41-60y	>60y		<16y	16-40y	41-60y	>60y	
**IFN-α**	2.03 (0.58)	1.37 (1.87)	0.35 (1.58)	0.07 (0.54)	<0.001	2.39 (0.79)	1.94 (1.80)	2.30 (2.28)	1.75 (2.00)	0.076
**IFN-β**	1.40 (0.49)	0.46 (1.43)	0.33 (1.05)	0.11 (0.35)	<0.001	1.43 (0.52)	1.65 (1.67)	1.60 (1.73)	1.35 (1.53)	0.821
**IFN-ε**	2.30 (0.43)	1.38 (1.85)	0.26 (1.72)	0.20 (0.70)	<0.001	2.43 (0.72)	2.39 (2.60)	0.90 (2.64)	1.11 (2.45)	0.037
**IFN-ω**	2.43 (1.04)	1.08 (2.42)	0.23 (1.81)	0.10 (0.75)	<0.001	2.75 (0.66)	2.87 (2.60)	1.72 (2.80)	1.80 (2.60)	0.067
**IFN-λ1**	0.06 (0.03)	0.06 (0.05)	0.02 (0.03)	0.01 (0.03)	<0.001	0.06 (0.02)	0.07 (0.04)	0.06 (0.05)	0.05 (0.05)	0.415
**IFN-λ2**	2.22 (0.52)	2.06 (2.82)	0.17 (1.52)	0.10 (0.29)	<0.001	2.16 (0.87)	2.50 (2.80)	1.09 (2.76)	1.32 (2.53)	0.170
**IFN-λ3**	4.38 (1.70)	2.32 (4.31)	0.90 (2.68)	0.52 (1.60)	<0.001	4.32 (0.90)	4.84 (1.84)	3.93 (2.47)	3.77 (1.45)	0.100
**ISG15**	1.44 (0.54)	2.06 (4.88)	1.92 (2.57)	1.20 (2.33)	0.049	1.87 (0.92)	3.42 (4.08)	5.30 (36.51)	7.70 (20.41)	<0.001
**ISG56**	0.51 (0.23)	0.52 (0.33)	0.36 (0.23)	0.23 (0.33)	<0.001	0.66 (0.23)	0.75 (0.95)	0.96 (2.68)	0.82 (3.20)	0.046

a Expression levels, calculated using 2^-ΔCt^, are indicated as median (interquartile range) of the group;

b p-values for comparisons among the age groups are calculated using Kruskal-Wallis tests.

### Basal Transcriptional Level of IFN Genes Is Elevated in Children and Decrease With Age

**Figure 2:**
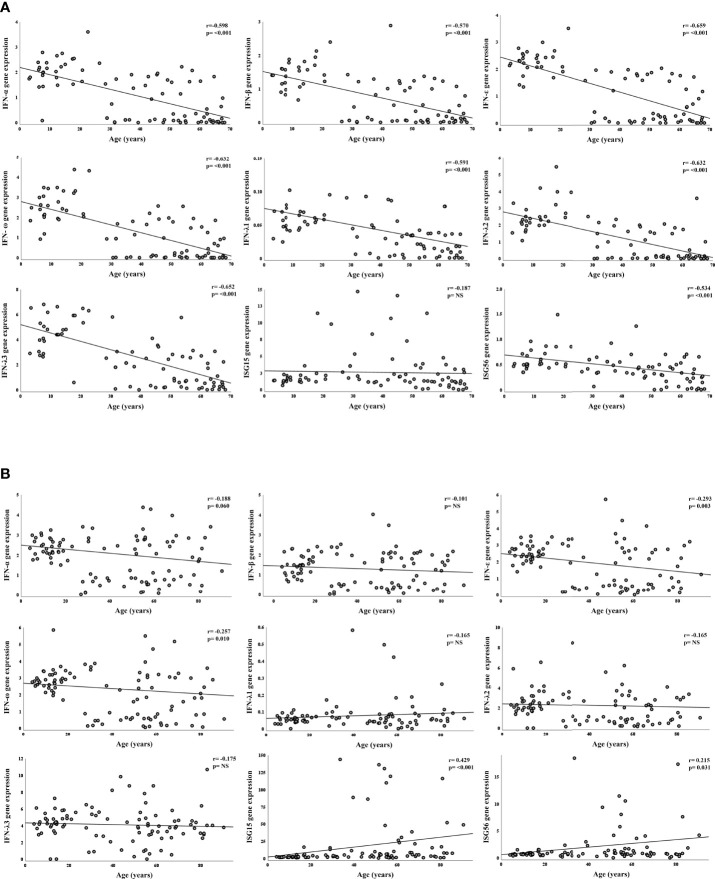
Correlation between gene expression and age, in SARS-CoV-2 negative and positive subjects. Relative mRNA expression values (y-axis) are calculated using the threshold cycle relative quantification (2^-ΔCt^); Spearman’s rho coefficient and p-values are reported in the upper right corner. *Panel A*: correlation of gene expression with age in years of SARS-CoV-2 negative subjects. *Panel B*: correlation of gene expression with age in years of SARS-CoV-2 positive subjects.

We first compared results from SARS-CoV-2 negative samples stratified by age groups (**Table 2**). All study genes, with the exception of ISG15, were far more expressed in children with progressively lower values found in young- and middle-aged adults and in the elderly (p<0.001). Uninfected young adults had levels of IFN-α, IFN-ε, IFN-ω, and IFN-λ3 transcripts at intermediate values between children and older adults; the level of IFN-β transcript was comparable to those found in the older adults’ groups, whereas IFN-λ1 and –λ2 were comparable to those in the children group (**Table 2**). These results were strengthened by the significant negative correlation between age and IFN-α, -β, -ε, -ω; the IFNs λ1-3; ISG56 (**Figure 2**). Contrastingly, ISG15 levels showed little difference among groups (p=0.049), with no significant correlation with age.

### IFN-ε and -ω are defective and ISGs Overexpressed in SARS-CoV-2 Infected Adults

The comparison of expression levels reached by IFN genes among SARS-CoV-2 positive age groups showed interesting differences. Regarding type I IFNs, transcripts of IFN-α and IFN-β did not differ among age groups (**Table 2**) and their values were not significantly correlated to age (**Figure 2**). Contrastingly, levels of IFN-ε and -ω were similarly high in children and young adults and much lower in the older age groups (p=0.007; p=0.021 in JT test for trends, respectively; **Table 2**) and were negatively correlated with age (**Figure 2B**). IFNs λ1-3 transcripts were comparable among the four age groups with no significant relationship with age (**Table 2**, **Figure 2B**). Notably, ISG56 and ISG15 were expressed at lower levels in the younger, and progressively increased with age (p=0.020; p<0.0001, in JT test for trends, respectively; **Table 2**); a moderate significant positive correlation between age and ISG56 and ISG15 was found (**Figure 2B**).

### IFN Genes Are Differentially Upregulated During SARS-CoV-2 Infection Depending on Age

**Figure 3:**
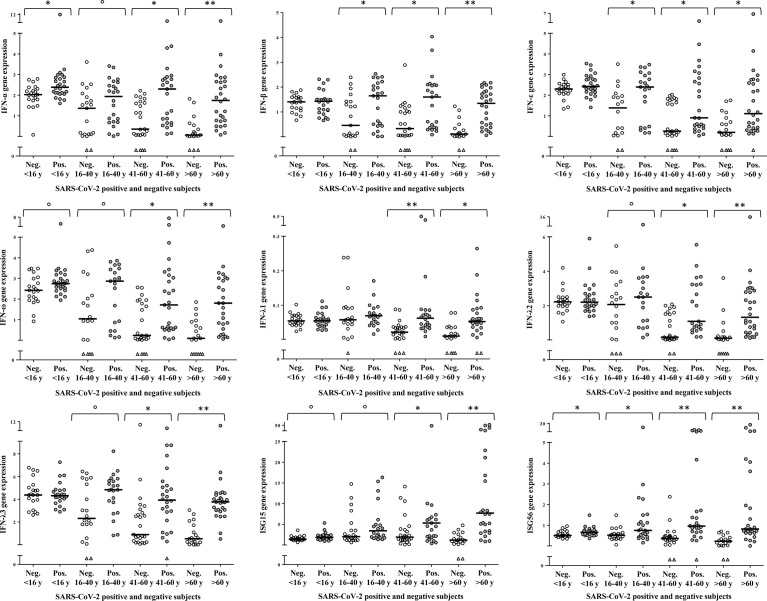
Age-specific activation of IFN genes. Relative mRNA expression values (y-axis), calculated using the threshold cycle relative quantification (2^-ΔCt^), are depicted as open circles for SARS-CoV-2 negative and as filled circles for SARS-CoV-2 positive samples, triangles indicate samples with Ct values >40. Horizontal lines represent the median value of the group indicated below the X-axis. P-values, calculated using Mann-Whitney tests for comparison between SARS-CoV-2 negative and positive samples of the same age group, are shown with symbols representing: °0.1<p>0.05, *0.05<p≥0.001, **p<0.001.

To dissect the age specific extent of activation of the type I/III IFN response to SARS-CoV-2 infection, a comparison between SARS-CoV-2 negative and -positive samples was performed within each age-group (**Figure 3**). In children, IFN-α (p=0.008) and ISG56 (p=0.012) were the only transcripts to be significantly higher in the SARS-CoV-2 positive with respect to the negative; there was a tendency to higher levels of IFN-ω (p=0.079) and ISG15 (p=0.057) whereas the other transcripts were comparable in SARS-CoV-2-positive and -negative children (**Figure 3**). In young adults, comparisons between SARS-CoV-2 negative and -positive samples showed a significant activation of IFN-β, IFN-ε and ISG56 (p=0.024; p=0.017; p=0.018, respectively) and a trend toward higher levels of IFN-α, IFN-ω, IFN-λ2, IFN-λ3 and ISG15 (p=0.078; p=0.100; p=0.078; p=0.084; p=0.064, respectively) in the SARS-CoV-2-positive with respect to the -negative whereas IFN-λ1 levels were comparable between groups (**Figure 3**). As regards the groups of middle-aged adults and older adults, all study genes were significantly activated in the SARS-CoV-2 positive subjects with respect to the negative of the same age group (p<0.005 for all comparisons, with the exception of ISG15 in the middle-aged adults, p=0.015; **Figure 3**).

It appears that children, starting from relatively high basal transcriptional levels, activate IFN genes comparatively less than adults upon SARS-CoV-2 infection, to reach transcript levels similar or higher with respect to the other age groups. In young adults, IFN genes’ basal expression and extent of transcription activation are intermediate between children and middle-aged/older adults; the latter groups are characterized by defective expression of IFN-ε and IFN-ω, and by higher ISGs transcription, upon SARS-CoV-2 infection.

### SARS-CoV-2 RNA Amount Was Related to the Level of ISG Transcription

SARS-CoV-2 diagnostic Ct values were obtained for 67/80 adults (83.75%) and were used as a proxy for viral load (a low Ct value reflects a high target RNA concentration) in relation to subjects’ data. Ct values did not differ (p=0.371) between males [mean ± standard deviation ( ± SD) = 27.9 ( ± 6.1)] and females [mean ( ± SD) = 26.3 ( ± 5.3)] nor among the three adults’ age groups (**Table 1**); Ct values recorded in samples from hospitalized patients [mean ( ± SD) = 27.9 ( ± 5.9)] were slightly higher than those in not-hospitalized adults [mean ± (SD) = 24.9 ( ± 5.6), p=0.093]. The level of expression of type I and III IFN-genes had no correlation with Ct values whereas a moderate negative correlation with ISG15 and ISG56 levels (r= -0.370, p=0.003; r= -0.342, p=0.007, respectively) was found.

### IFN Coding Genes Are More Expressed in Symptomatic Children and in Hospitalized Adults

**Figure 4:**
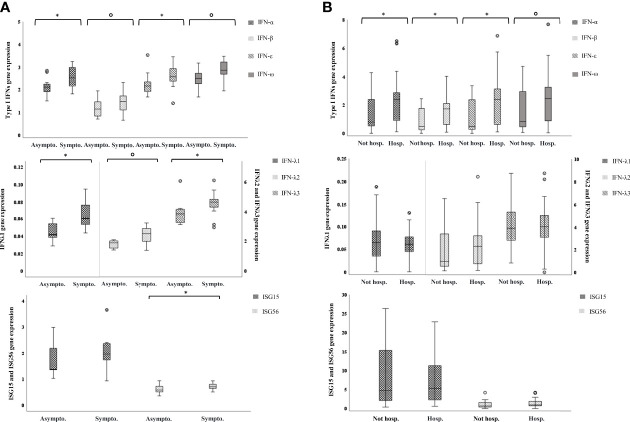
IFN genes are more expressed in symptomatic children and in hospitalized adults. Relative mRNA expression values (y-axis), calculated using the threshold cycle relative quantification (2^-ΔCt^), are shown in box plot graphs: the box represents the lower and upper quartiles, the line in the box represents the median, the whiskers show the lowest and highest values, and the outliers are represented by open circles. Above box plot pairs, p-values calculated using Mann-Whitney tests for comparisons are shown with symbols representing: °0.1<p>0.05, *0.05<p≥0.001. **(A)** IFN genes expression in asymptomatic vs symptomatic children. **(B)** IFN genes expression in not-hospitalized vs hospitalized adults.

Among SARS-CoV-2 positive children, 11 were completely asymptomatic and 15 had fever and/or respiratory symptoms; none required hospitalization (**Table 1**). There was no difference in age and sex between asymptomatic and symptomatic children (data not shown). Type I IFN genes were slightly more expressed in children presenting symptoms with respect to the asymptomatic (p-values for comparisons between the two groups of IFN-α, -β, -ε, and -ω are: p=0.032, p=0.087, p=0.069, p=0.032, respectively, **Figure 4A**); type III IFN transcripts were far higher in the symptomatic group (p-values for IFN-λ1, -λ2, -λ3 are: p=0.001, p=0.020, p=0.009, respectively, **Figure 4A**). Contrarily, ISG56 levels were higher in the asymptomatic, p=0.047) and ISG15 levels were comparable in the two groups (**Figure 4A**).

Nearly all adult patients experienced fever and respiratory symptoms (77/80, 96.25%), 27/80 (33.75%) had a mild COVID-19 disease and were observed at home whereas 53 were hospitalized (66.25%); none was admitted to an intensive care unit or died (**Table 1**). Hospitalized patients were significantly older (p<0.001) than those not hospitalized but did not differ by sex (**Table 1**). When analyzing transcripts levels in relation to the need of hospitalization, we observed a significant up-regulation of type I IFNs, IFN-α, -β, -ε, and a trend of higher levels for IFN-ω as well, in the hospitalized adults (p=0.008, p=0.005, p=0.005, p=0.057, respectively); the IFNs λ1-3 and the ISGs were not different between the two groups (**Figure 4B**).

## Discussion

Recent studies proposed that the mild course of SARS-CoV-2 infections in children is associated with their ability to mount an early and effective antiviral response (18, 19, 23, 24). Their common finding was pre-activated levels of innate immunity genes in the nasal epithelium of healthy children compared to adults (18, 19, 23, 24). Our results confirm that IFN basal levels are up-regulated in children comparing with adults of different age groups, provide further insights into this response in the, less-studied, young adults group, and novel findings regarding IFN-ε and IFN-ω expression.

We detected elevated basal expression of type I/III IFN genes in children and observed age-related decrease of their expression. In particular, basal expression of type I IFNs in young adults was intermediate (IFN-α, IFN-ε and IFN-ω) with respect to pediatric and older adults ages, or similar to that in older adults (IFN-β); among type III IFNs, basal expression of IFN-λ1 and -λ2 was similar between children and young adults whereas IFN-λ3 values in the young were intermediate to those found in the other groups. Collectively, these differences in basal transcriptional levels suggest that, in the NP mucosa, IFN types are differently regulated by age.

Upon SARS-CoV-2 infection, children upregulated transcription of IFN-α and ISG56, and moderately also IFN-ω and ISG15; young adults showed moderate transcriptional activation of all study genes with the exception of IFN-λ1, whereas all study genes were significantly upregulated at older ages. Considering that transcript levels of the IFNs coding genes showed more differences with age in healthy controls than in infected individuals, it is plausible to speculate that IFNs are produced during SARS-CoV-2 infection at any age but are more readily available in the respiratory mucosa at younger ages than in the older. This scenario is coherent with the current view that pre-activated levels of the IFN genes are essential to a fast and effective anti-SARS-CoV-2 response as seen in children and in young adults (18, 19, 24) whereas delayed and sustained IFN activation may be detrimental to the respiratory mucosa and associated to the worsening of respiratory symptoms (4). In accordance with this hypothesis, we observed that several IFN genes reached higher levels in children presenting symptoms with respect to the asymptomatic, and in hospitalized patients with respect to not-hospitalized adults.

Coherently with our findings, in the more recent and larger transcriptome study (24) of cells from NP swabs, the most striking difference by age in gene expression signature was observed in the IFN-α signaling that was pre-activated in not-infected children and only slightly induced after SARS-CoV-2 infection; adults (not differentiated by age in that study) showed very low basal levels of IFN-α related genes but these were strongly activated upon infection (24). In contrast with our data, Yoshida et al. (24) found, in adults’ epithelial cells, a higher IFN-α in mild COVID-19 compared to severe disease. In this regard, it should be underlined that while a protective role of IFNs in the first phases of SARS-CoV-2 replication in the nasal mucosa is well documented, the issue of a favorable or detrimental role of sustained IFN production in older hospitalized patients is still debated and conflicting results have been published on comparative levels of IFNs and ISGs induction found in mild vs severe COVID-19 (13, 14, 20, 25).

Remarkably, we detected age-related differences, not previously reported, also in the expression of IFN-ε and IFN-ω.

IFN-ϵ is dissimilar from other type I IFNs mainly due its regulation; its expression is constitutively high mainly in the epithelial cells of the female reproductive tract (26). In addition, *in vitro* studies demonstrated its ability to restrict HIV replication in epithelial cells, monocyte derived macrophages and T cells (26, 27); in mouse lung, IFN-ϵ is constitutively expressed and able to stimulate an antiviral response upon infection (28) but no such data have been published on IFN-ϵ expression in human respiratory mucosal cells. Thus, we showed for the first time in NP cells, higher basal levels of IFN-ϵ in children compared to adults with no difference according to sex. Interestingly, upon SARS-CoV-2 infection, IFN-ϵ activation was more intense in young adults, reaching levels observed in SARS-CoV-2 positive children If confirmed in a larger number of cases, IFN-ϵ elevated levels at younger ages may suggest its possible role with regards to a successful antiviral response. On the other hand, as seen for the other type I IFNs, more IFN-ϵ was expressed in those who were hospitalized, leading to detrimental effects; interestingly, IFN-ε expression can be upregulated by TNF-α, one of the pro-inflammatory cytokine commonly upregulated in the course of COVID-19 (29).

As regards IFN-ω expression at baseline and upon SARS-CoV-2 infection, differences by age were similar to those seen for IFN-ϵ. Even in the absence of experimental evidences, a potential anti-SARS-CoV-2 activity of IFN-ω was hypothesized (15) based on detection of anti-IFN-ω auto-Abs in a fraction of severe COVID-19 patients together with anti IFN-α2 auto-Abs (10, our submitted data). IFN-ω shares ∼60% amino acid similarity to IFN-α, the type I IFN more phylogenetically close; in humans only one IFN-ω is produced compared to the 13 IFN-α subtypes (30). Intriguingly, IFN-ω is still diversifying in many species, including bats and pigs and protect them from excessive tissue damage following infection by several viruses, including Coronaviridae and Orthomixoviridae that can cause severe diseases in humans (30, 31). Further data are needed to confirm IFN-ω induction in respiratory mucosal cells and its possible role as an anti-SARS-CoV-2 weapon in our innate responses.

As regards type III IFNs, we found that their expression was higher in children at baseline as well as upon infection; their levels were not related to SARS-CoV-2 viral load nor to the occurrence of symptoms in children nor with hospitalization in adults. Specifically, IFN-λ1 relative expression was very low compared to that of IFN-λ2 and -λ3 in all tested samples but the age-related differences in SARS-CoV-2-negative and -positive samples and their relative levels of activation were similar among type III IFNs. In the study by Sposito et al, IFN-λ1 was the more potent ISGs inducer with respect to λ2 and - λ3 and to type I IFNs in mild COVID-19 adults’ patients; IFN-λ1 potency could be a reason to keep its basal expression and its activation low upon infections. In fact, besides being a first line defense against mucosal pathogens, type III IFNs can also exert harmful effects during respiratory viral infections including SARS-CoV-2, in particular by lowering lung epithelial repair (32–34). Indeed, a well-balanced baseline expression of type I/III IFNs’ is involved in the physiological regulation of anti-microbial response in the intestinal, cervical and respiratory mucosa (35–37). A scenario is emerging in which coordinated, quick and effective antiviral actions, relying on pre-activated IFNs’ expression, can halt SARS-CoV-2 replication in the first days post-infection in the nasal mucosa, leading to asymptomatic or mild course whereas a later response, triggering high and persistent expression of antiviral genes, may be less effective and cause respiratory damage (14–19, 24, 38).

As concerns basal ISG expression, ISG56 level was higher in children and young adults than in older adults while ISG15 level was lower only in the elderly. Upon SARS-CoV-2 infection, the two ISGs reached far higher levels in adults, coherently with the overall lower IFN activation at younger ages than in adults. Moreover, ISGs transcripts were related to the amount of SARS-CoV-2 RNA as estimated by the diagnostic Ct value in adult patients. The latter finding agrees with previous studies finding a relationship between ISG combined levels and viral loads (11, 17, 21); our analysis focused only on two of the very numerous ISG stimulated by type I and III IFNs and also by IFN-independent pathways and cannot add much to this issue. Nonetheless, the biological value of testing two ISGs only is strengthened by transcriptomic analysis reporting that ISGs expression was co-regulated in SARS-CoV-2 infected respiratory cells (11, 17, 19).

Our study has several limitations: no information on days since SARS-CoV-2 infection; lack of follow-up samples; the relatively low amount of NP cells obtained by residual diagnostic specimens that limited the number of measurements. Regarding this last point, it may have been informative to measure the expression of Pattern Recognition Receptors genes and those triggered in their signaling cascade, as these genes were shown to be up-regulated in children with respect to adults (19). Another limitation due to low amount of NP specimens is that we could not evaluate cell types composition. Indeed, the relative abundance of resident immune cells in the NP mucosa is higher in the younger compared to adults (39); accordingly, the pre-activated innate immune response in children may be due to differential transcriptional programs in epithelial cells and/or in immune cells. In this study, activation of the IFN-response was measured on mixed NP cells and this method may appear basic and less informative with respect to state of the art single cell RNA sequencing techniques. Nevertheless, Real time PCR assays performed with gene-specific probes are reliable, not-expensive, quantitative tests that allowed to analyze nearly two hundred of residual diagnostic samples from different age groups and add to the existing knowledge.

In conclusion, we could confirm in NP samples including all ages, higher basal levels of type I and III IFNs in children and suggest that the IFN response is partially pre-activated also in young adults.

Moreover, we recognized a possible anti-SARS-CoV-2 role of the less well-characterized IFN-ϵ and IFN-ω, that should be further investigated *in vitro* and *in vivo*. On the other hand, IFN-ϵ and IFN-ω seem to be no exception to the rule that IFNs may also exert detrimental roles and tissue damage. These data encourage further investigation on the possible protective effects of several different IFN types against severe COVID-19.

## Data Availability Statement

The original contributions presented in the study are included in the article/supplementary files, further inquiries can be directed to the corresponding author.

## Ethics Statement

The studies involving human participants were reviewed and approved by Institutional Review board and the Ethics Committee (Policlinico Umberto I Hospital, Sapienza, University of Rome, Rif. 5836, Prot. 0690/2021. Written informed consent to participate in this study was provided by the participants’ legal guardian/next of kin.

## Author Contributions

Conceptualization: CS, and AP. Methodology: AP and RN. Formal analysis: GC, LM and LP. Investigation: FF, MF, AV, LS and GO. Data acquisition and analysis: MG, FM, GD’E. Writing, review and editing: AP and CS. Supervision: AP, GA. Funding acquisition, GA, CS. All authors have read and agreed to the published version of the manuscript.

## Funding

This research was funded by a grant from the Italian Ministry of Health (COVID-2020-12371817) to GA. A grant from Sapienza University (ATENEO H2020, PH120172B4BA8CAF) to GA. A grant from Sapienza University (RM12117A513C1DDD) to CS. Funding sources were not involved in study design; in the collection, analysis and interpretation of data; in the writing of this manuscript; and in the decision to submit the article for publication.

## Conflict of Interest

The authors declare that the research was conducted in the absence of any commercial or financial relationships that could be construed as a potential conflict of interest.

## Publisher’s Note

All claims expressed in this article are solely those of the authors and do not necessarily represent those of their affiliated organizations, or those of the publisher, the editors and the reviewers. Any product that may be evaluated in this article, or claim that may be made by its manufacturer, is not guaranteed or endorsed by the publisher.

## References

[1] Castagnoli R Votto M Licari A Brambilla I Bruno R Perlini S. Severe Acute Respiratory Syndrome Coronavirus 2 (SARS-CoV-2) Infection in Children and Adolescents: A Systematic Review. JAMA Pediatr (2020) 174:882–9. doi: 10.1001/jamapediatrics.2020.1467 32320004

[2] Cristiani L Mancino E Matera L Nenna R Pierangeli A Scagnolari C. Will Children Reveal Their Secret? The Coronavirus Dilemma. Eur Respir J (2020) 23;55:2000749. doi: 10.1183/13993003.00749-2020 PMC7113798 32241833

[3] Bogunovic D Merad M. Children and SARS-CoV-2. Cell Host Microbe (2021) 29:1040–2. doi: 10.1016/j.chom.2021.06.015 PMC8279573 34265242

[4] Kim YM Shin EC. Type I and III Interferon Responses in SARS-CoV-2 Infection. Exp Mol Med (2021) 53:750–60. doi: 10.1038/s12276-021-00592-0 PMC8099704 33953323

[5] Schultze JL Aschenbrenner AC. COVID-19 and the Human Innate Immune System. Cell (2021) 184:1671–92. doi: 10.1016/j.cell.2021.02.029 PMC7885626 33743212

[6] Lowery SA Sariol A Perlman S. Innate Immune and Inflammatory Responses to SARS-CoV-2: Implications for COVID-19. Cell Host Microbe (2021) 29:1052–62. doi: 10.1016/j.chom.2021.05.004 PMC8126603 34022154

[7] Clementi N Ferrarese R Criscuolo E Diotti RA Castelli M Scagnolari C. Interferon-β-1a Inhibition of Severe Acute Respiratory Syndrome-Coronavirus 2 *In Vitro* When Administered After Virus Infection. J Infect Dis (2020) 222:722–5. doi: 10.1093/infdis/jiaa350 PMC7337790 32559285

[8] Felgenhauer U Schoen A Gad HH Hartmann R Schaubmar AR Failing K. Inhibition of SARS-CoV-2 by Type I and Type III Interferons. J Biol Chem (2020) 295:13958–64. doi: 10.1074/jbc.AC120.013788 PMC7549028 32587093

[9] Zhang Q Bastard P Liu Z Le Pen J Moncada-Velez M Chen J. Inborn Errors of Type I IFN Immunity in Patients With Life-Threatening COVID-19. Science (2020) 370:eabd4570. doi: 10.1126/science.abd4570 32972995 PMC7857407

[10] Bastard P Rosen LB Zhang Q Michailidis E Hoffmann HH Zhang Y. Autoantibodies Against Type I IFNs in Patients With Life-Threatening COVID-19. Science (2020) 370:eabd4585. doi: 10.1126/science.abd4585 32972996 PMC7857397

[11] Lopez J Mommert M Mouton W Pizzorno A Brengel-Pesce K Mezidi M. Early Nasal Type I IFN Immunity Against SARS-CoV-2 Is Compromised in Patients With Autoantibodies Against Type I IFNs. J Exp Med (2021) 218:e20211211. doi: 10.1084/jem.20211211 34357402 PMC8352718

[12] Blanco-Melo D Nilsson-Payant BE Liu WC Uhl S Hoagland D Møller R. Imbalanced Host Response to SARS-CoV-2 Drives Development of COVID-19. Cell (2020) 181:1036–1045.e9. doi: 10.1016/j.cell.2020.04.026 32416070 PMC7227586

[13] Zhou Z Ren L Zhang L Zhong J Xiao Y Jia Z. Heightened Innate Immune Responses in the Respiratory Tract of COVID-19 Patients. Cell Host Microbe (2020) 27:883–890.e2. doi: 10.1016/j.chom.2020.04.017 32407669 PMC7196896

[14] Sposito B Broggi A Pandolfi L Crotta S Clementi N Ferrarese R. The Interferon Landscape Along the Respiratory Tract Impacts the Severity of COVID-19. Cell (2021) 184:4953–4968.e16. doi: 10.1016/j.cell.2021.08.016 34492226 PMC8373821

[15] Decker T. The Early Interferon Catches the SARS-CoV-2. J Exp Med (2021) 218:e20211667. doi: 10.1084/jem.20211667 34424267 PMC8404473

[16] Lieberman NAP Peddu V Xie H Shrestha L Huang ML Mears MC. *In Vivo* Antiviral Host Transcriptional Response to SARS-CoV-2 by Viral Load, Sex, and Age. PloS Biol (2020) 18:e3000849. doi: 10.1371/journal.pbio.3000849 32898168 PMC7478592

[17] Cheemarla NR Watkins TA Mihaylova VT Wang B Zhao D Wang G. Dynamic Innate Immune Response Determines Susceptibility to SARS-CoV-2 Infection and Early Replication Kinetics. J Exp Med (2021) 218:e20210583. doi: 10.1084/jem.20210583 34128960 PMC8210587

[18] Pierce CA Sy S Galen B Goldstein DY Orner E Keller MJ. Natural Mucosal Barriers and COVID-19 in Children. JCI Insight (2021) 6:e148694. doi: 10.1172/jci.insight.148694 PMC8262299 33822777

[19] Loske J Röhmel J Lukassen S Stricker S Magalhães VG Liebig J. Pre-Activated Antiviral Innate Immunity in the Upper Airways Controls Early SARS-CoV-2 Infection in Children. Nat Biotechnol (2021) 49:3197–24. doi: 10.1038/s41587-021-01037-9 34408314

[20] Gilbert C Lefeuvre C Preisser L Pivert A Soleti R Blanchard S. Age-Related Expression of IFN-λ1 Versus IFN-I and Beta-Defensins in the Nasopharynx of SARS-CoV-2-Infected Individuals. Front Immunol (2021) 12:750279. doi: 10.3389/fimmu.2021.750279 34858406 PMC8631500

[21] Scagnolari C Pierangeli A Frasca F Bitossi C Viscido A Oliveto G. Differential Induction of Type I and III Interferon Genes in the Upper Respiratory Tract of Patients With Coronavirus Disease 2019 (COVID-19). Virus Res (2021) 295:198283. doi: 10.1016/j.virusres.2020.198283 33418027 PMC7834390

[22] Pierangeli A Gentile M Di Marco P Pagnotti P Scagnolari C Trombetti S. Detection and Typing by Molecular Techniques of Respiratory Viruses in Children Hospitalized for Acute Respiratory Infection in Rome, Italy. J Med Virol (2007) 79:463–8. doi: 10.1002/jmv.20832 PMC7166338 17311326

[23] Koch CM Prigge AD Anekalla KR Shukla A Do Umehara HC Setar L. Age-Related Differences in the Nasal Mucosal Immune Response to SARS-CoV-2. Am J Respir Cell Mol Biol (2022) 66(2):206–22. doi: 10.1165/rcmb.2021-0292OC PMC8845137 34731594

[24] Yoshida M Worlock KB Huang N Lindeboom RGH Butler CR Kumasaka N. Local and Systemic Responses to SARS-CoV-2 Infection in Children and Adults. Nature (2021) 602(198283):321–27 doi: 10.1038/s41586-021-04345-x PMC8828466 34937051

[25] Lucas C Wong P Klein J Castro TBR Silva J Sundaram M. Longitudinal Analyses Reveal Immunological Misfiring in Severe COVID-19. Nature (2020) 584:463–9. doi: 10.1038/s41586-020-2588-y PMC7477538 32717743

[26] Marks ZRC Campbell N deWeerd NA Lim SS Gearing LJ Bourke NM. PROPERTIES AND FUNCTIONS OF THE NOVEL TYPE I INTERFERON EPSILON. Semin Immunol (2019) 43:101328. doi: 10.1016/j.smim.2019.101328 31734130

[27] Tasker C Subbian S Gao P Couret J Levine C Ghanny S. IFN-ϵ Protects Primary Macrophages Against HIV Infection. JCI Insight (2016) 1(20):e88255. doi: 10.1172/jci.insight.88255 27942584 PMC5135270

[28] Xi Y Day SL Jackson RJ Ranasinghe C. Role of Novel Type I Interferon Epsilon in Viral Infection and Mucosal Immunity. Mucosal Immunol (2012) 5(6):610–22. doi: 10.1038/mi.2012.35 PMC3481022 22617838

[29] Matsumiya T Prescott SM Stafforini DM. IFN-Epsilon Mediates TNF-Alpha-Induced STAT1 Phosphorylation and Induction of Retinoic Acid-Inducible Gene-I in Human Cervical Cancer Cells. J Immunol (2007) 179(7):4542–9. doi: 10.4049/jimmunol.179.7.4542 17878351

[30] Shields LE Jennings J Liu Q Lee J Ma W Blecha F. Cross-Species Genome-Wide Analysis Reveals Molecular and Functional Diversity of the Unconventional Interferon-ω Subtype. Front Immunol (2019) 10:1431. doi: 10.3389/fimmu.2019.01431 31293589 PMC6603160

[31] Fox LE Locke MC Lenschow DJ. Context Is Key: Delineating the Unique Functions of Ifnα and Ifnβ in Disease. Front Immunol (2020) 11:606874. doi: 10.3389/fimmu.2020.606874 33408718 PMC7779635

[32] Broggi A Granucci F Zanoni I. Type III Interferons: Balancing Tissue Tolerance and Resistance to Pathogen Invasion. J Exp Med (2020) 217(1):e20190295. doi: 10.1084/jem.20190295 31821443 PMC7037241

[33] Major J Crotta S Llorian M McCabe TM Gad HH Priestnall SL. Type I and III Interferons Disrupt Lung Epithelial Repair During Recovery From Viral Infection. Science (2020) 369(6504):712–7. doi: 10.1126/science.abc2061 PMC7292500 32527928

[34] Broggi A Ghosh S Sposito B Spreafico R Balzarini F Lo Cascio A. Type III Interferons Disrupt the Lung Epithelial Barrier Upon Viral Recognition. Science (2020) 369(6504):706–12. doi: 10.1126/science.abc3545 PMC7292499 32527925

[35] Kawashima T Kosaka A Yan H Guo Z Uchiyama R Fukui R. Double-Stranded RNA of Intestinal Commensal But Not Pathogenic Bacteria Triggers Production of Protective Interferon-β. Immunity (2013) 38:1187–97. doi: 10.1016/j.immuni.2013.02.024 23791646

[36] Fung KY Mangan NE Cumming H Horvat JC Mayall JR Stifter SA. Interferon-ϵ Protects the Female Reproductive Tract From Viral and Bacterial Infection. Science (2013) 339:1088–92. doi: 10.1126/science.1233321 PMC3617553 23449591

[37] Lazear HM Nice TJ Diamond MS. Interferon-λ: Immune Functions at Barrier Surfaces and Beyond. Immunity (2015) 43:15–28. doi: 10.1016/j.immuni.2015.07.001 26200010 PMC4527169

[38] Hatton CF Botting RA Dueñas ME Haq IJ Verdon B Thompson BJ. Delayed Induction of Type I and III Interferons Mediates Nasal Epithelial Cell Permissiveness to SARS-CoV-2. Nat Commun (2021) 12:7092. doi: 10.1038/s41467-021-27318-0 34876592 PMC8651658

[39] Winkley K Banerjee D Bradley T Koseva B Cheung WA Selvarangan R. Immune Cell Residency in the Nasal Mucosa May Partially Explain Respiratory Disease Severity Across the Age Range. Sci Rep (2021) 11:15927. doi: 10.1038/s41598-021-95532-3 34354210 PMC8342554

